# ^61^Cu-Labeled Radiotracers: Alternative or Choice?

**DOI:** 10.2967/jnumed.123.266171

**Published:** 2023-12

**Authors:** Melpomeni Fani, Guillaume P. Nicolas

**Affiliations:** 1 Division of Radiopharmaceutical Chemistry, University Hospital Basel, Basel, Switzerland; and; 2 Division of Nuclear Medicine, University Hospital Basel, Basel, Switzerland

The pursuit of the perfect radionuclide for imaging or therapy is ongoing, as no single choice can universally meet all applications. The field of nuclear medicine has seen significant advancements in radiotheranostics, particularly in neuroendocrine tumors (e.g., [^68^Ga]Ga/[^177^Lu]Lu-DOTATATE) and prostate cancer (e.g., [^68^Ga]Ga-prostate-specific membrane antigen [PSMA]–11, [^18^F]F-DCFPyL, and [^177^Lu]Lu-PSMA-617) (1). In recent years, numerous new radiopharmaceuticals and radionuclides have emerged (1). Factors such as rising demand, costs, and availability must be considered when selecting a radionuclide for clinical use. To maximize benefits, it is also crucial to ensure compatibility between the radionuclide’s physical properties and the ligand’s pharmacokinetic properties, such as biologic half-life.

Copper radioisotopes, namely ^61^Cu and ^64^Cu for PET imaging and ^67^Cu for therapy, are highly desirable because of their suitability for their respective applications (2*,*^3^). ^61/64^Cu/^67^Cu offers a superior theranostic match compared with the commonly used ^68^Ga/^177^Lu pair, thanks to chemically identical structures shared between the imaging and therapeutic radiotracers (^x^Cu-ligand). Of note, a few alternatives also have this elementally matched pair attribute, such as the emerging pairs ^203^Pb/^212^Pb and ^152^Tb/^161^Tb. This allows for consistent biodistribution and pharmacokinetics, essential for precise pretherapeutic dosimetry. Among copper radioisotopes, ^64^Cu is commonly used for PET imaging because of its longer half-life (12.7 h) and commercial availability. In 2020, [^64^Cu]Cu-DOTATATE received approval, and clinical trials are evaluating various ^64^Cu-labeled PSMAs.

## WHY ^61^CU VERSUS OTHER PET RADIONUCLIDES?

**FIGURE 1.**
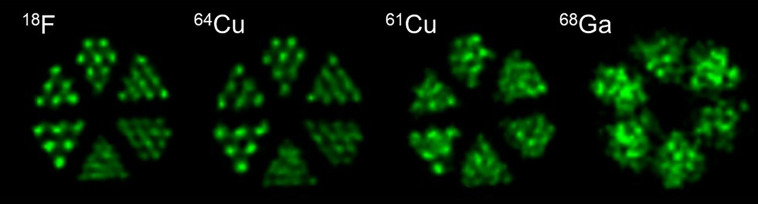
Visual comparison of spatial resolution of different PET radionuclides using Jaszczak phantom, 0.7–1.2 mm, filled with 6.5 MBq of [^18^F]FDG (^18^F), 13 MBq of [^64^Cu]CuCl_2_ (^64^Cu), 5.9 MBq of [^61^Cu]CuCl_2_ (^61^Cu), and 6 MBq of [^68^Ga]GaCl_3_ (^68^Ga). PET images were obtained within 30 min on small-animal PET scanner (β-CUBE; Molecubes) and reconstructed using ordered-subsets maximization expectation (slice thickness, 0.4 mm).

In Table 1, the physical properties of ^61^Cu are compared with other established PET radionuclides, and Figure 1 illustrates the PET image resolution using a phantom. ^61^Cu (half-life, 3.33 h; 61% β^+^-fraction; mean positron energy [E_β_^+^], 500 keV; maximum E_β_^+^, 1,216 keV) exhibits more favorable characteristics than ^64^Cu. Even though ^64^Cu has a lower positron energy (maximum E_β_^+^, 655 keV) with intrinsically better spatial image resolution, ^61^Cu has a higher number of positrons (β^+^) emitted per decay (61% compared with 17.9%), leading to improved sensitivity, as already indicated by the pioneer work of McCarthy et al. (4). This provides the opportunity for a lower injected activity or a shorter scanning time to achieve adequate photon count statistics. Furthermore, the shorter half-life and absence of β^−^ particles (which account for 39% of decays in ^64^Cu) result in a reduced radiation dose to the patient. In daily clinical practice, using ^61^Cu may offer greater convenience for patient management, especially in countries with stricter radioprotection regulations.

**TABLE 1.** Physical Properties of ^61^Cu vs. Commonly Used PET Radionuclides

Physical property	^61^Cu	^64^Cu	^68^Ga	^18^F
Half-life (h)	3.33	12.7	1.13	1.83
Decay, yield (%)	β^+^ 61	β^+^ 17.9	β^+^ 88.9	β^+^ 96.7
	EC 39	EC 43.5	EC 11.1	EC 3.3
		β^+^ 39.0		
Eβ^+^ (keV)				
Maximum	1,216	653	1,899	635
Mean	500	278	830	250
β^+^ range in water (mm)				
Maximum	5.2	2.5	9.6	2.4
Mean	1.3	0.7	2.4	0.6

EC = electron capture.

Because of its intermediate half-life between ^68^Ga (68 min) and ^64^Cu (12.7 h), ^61^Cu enables PET scans to be conducted 3–6 h after injection, offering improved diagnostic performance due to higher image contrast and tumor-to-background ratios over time, compared with ^68^Ga-labeled tracers. This can enhance sensitivity and accuracy while avoiding false-positive signals. In addition, multiple-time-point scans enable pretherapeutic dosimetry estimations. ^61^Cu-labeled tracers are less susceptible to delays that may occur after administration to a patient than are ^68^Ga-labeled tracers. Certainly, in daily routine the 24-h availability of the generator-produced ^68^Ga is convenient, and scans at early time points are preferable. Nevertheless, the limited production capacity of the ^68^Ga tracers (2–3 patient doses) raises certain concerns, and multiple serial production is hampered by the waiting time between 2 consecutive elutions of the generator. ^61^Cu brings more flexibility in performing radiosynthesis, shipping from a central producer to satellite institutions, and scheduling patients, especially because of its longer half-life.

Although ^61^Cu does not possess better physical properties than ^18^F, it does offer advantageous chemical properties due to its ability to be labeled using chelators. As a result, radiosynthesis for ^61^Cu is simple, allowing for kit (shake-and-bake) formulation without requiring expensive infrastructure such as module-assisted radiosynthesis or purification systems commonly used for ^18^F radiotracers. These qualities make it well suited for daily routine use. Moreover, the notable structural differences between chelator-based therapeutics and ^18^F-based PET tracers bear a high risk of variations in biodistribution between the diagnostic and the therapeutic radiotracer. The increasing adoption of chelators, such as the NOTA chelator used in the Al^18^F strategy, in developing ^18^F-labeled tracers is a step toward solving this disparity. ^61^Cu offers the possibility of a chemically identical therapeutic companion (^67^Cu-labeled tracer) or a similar one (e.g., [^64^Cu]Cu/[^177^Lu]Lu-DOTATATE, in analogy to [^68^Ga]Ga/[^177^Lu]Lu-DOTATATE).

Copper chemistry is widely understood and straightforward (2*,*^3^*,*^5^). However, the challenge in developing ^x^Cu-based radiotracers lies in the in vivo stability of the ^x^Cu-chelator complex (3). This challenge is due to the risk of ^x^Cu(II) decomplexation (e.g., transchelation or transmetallation) (5) and the bioreduction of ^x^Cu(II)/^x^Cu(I). ^x^Cu(I) may be released from the chelator and incorporated into endogenous copper-binding proteins, followed by accumulation in the liver and other off-target tissues. Thus, a range of chelators has been specifically designed for ^x^Cu-based radiotracers (5). In contrast to the widely used DOTA and its derivatives, which demonstrate the in vivo instability of the copper-DOTA complex, chelators such as sarcophagine and NODAGA have shown promise in circumventing this issue. Additionally, these ^x^Cu-tailored chelators offer the advantage of room temperature labeling within a few minutes (shake-no-bake approach), making the production of radiotracers even faster and simpler.

## WHICH LIGANDS MAY BENEFIT FROM ^61^CU?

The favorable properties of ^61^Cu make it suitable for delayed imaging with ligands that exhibit peak tumor uptake 1–2 h after injection and have fast body clearance. This is especially relevant for small molecules or peptides. Indicative ligands in combination with ^61^Cu are somatostatin analogs (SSA), which have a peak tumor uptake of between 4 and 24 h. Currently, ^68^Ga-SSA PET/CT imaging is acquired 45–90 min after injection, which might be suboptimal. Thus, delayed imaging using ^61^Cu could better exploit the pharmacokinetic properties of SSA and further enhance image contrast and sensitivity. However, the benefit of delayed imaging with [^64^Cu]Cu-DOTATATE compared with ^68^Ga-SSA PET or ^18^F-SSA PET scans at 1 h after injection is still uncertain (6*,*^7^).

In PSMA scans using ^68^Ga-PSMA ligands, an uptake time of approximately 60 min is recommended. Yet, PET/CT imaging at 3 h after injection has demonstrated improved detection of tumor lesions with higher uptake and contrast (8). For unclear findings, particularly for lesions near the bladder or ureter, or in bone scans using [^18^F]PSMA-1007, scanning at a later time point may be considered (9).

Using ^61^Cu in exendin-4 PET imaging would be beneficial because of difficulties associated with module-assisted radiolabeling and elevated temperatures. Fast radiolabeling at room temperature may resolve these issues, whereas late scanning (>2.5 h after injection) enables washout of exendin-4 from the duodenum, pancreas, and kidneys. This may lead to more conclusive findings and may reveal small insulinomas in the tail of the pancreas that were obscured on earlier scans because of the high renal uptake of the radiotracer (10*,*^11^).

## WHERE DO ^61^CU PRODUCTION, DISTRIBUTION, AND AVAILABILITY STAND?

Despite its favorable physical properties, the development of ^61^Cu has been limited by availability constraints. Recently, 2 methods for large-scale production of ^61^Cu using liquid (12) and solid (13) targets have been developed for commercial production on a standard medical cyclotron (16.5- or 18-MeV proton capability). The main routes of production involve proton bombardment of inexpensive natural zinc, or enriched ^64^Zn or ^61^Ni and deuteron bombardment of natural nickel or enriched ^60^Ni. Although liquid targets offer easy processing, their lower production yields and longer irradiation times may restrict widespread use, especially in view of interference with the routine ^18^F production. Yet, simultaneous production of ^61^Cu and ^18^F is possible using a dual-proton-beam setup. On the other hand, solid target production through an 8.4-MeV deuteron bombardment of ^nat^Ni or ^60^Ni or a 12- to 14-MeV proton bombardment of ^61^Ni required shorter times and can be scaled from 4 to 60 GBq by increasing the enrichment levels of the ^x^Ni. However, solid targets require additional steps such as target material dissolution but provide higher production yields at shorter irradiation times and scalability based on material enrichment. The choice between liquid and solid targets depends on production requirements, material availability, and commercial demand. A comparison of the production routes is listed in Table 2.

**TABLE 2.** Comparison of Production Routes for ^61^Cu

Target	Target material	Target material cost/mg	Nuclear reaction	Target concentration or weight	Beam current (μA)	Irradiation time (min)	Activity produced (GBq)
Liquid	^nat^Zn	$0.003	^nat^Zn(p,α)^61^Cu	200 mg/mL	70	180	1.8 ± 0.2
Liquid	^64^Zn	$0.5–0.7	^64^Zn(p,α)^61^Cu	200 mg/mL	70	180	3.3 ± 0.4
Solid	^nat^Ni	$0.005	^nat^Ni(d,n)^61^Cu	80 mg	60	60	2.0 ± 0.2
Solid	^61^Ni	$20–25	^61^Ni(p,n)^61^Cu	50 mg	40	30	3.5 ± 0.5

There are several major factors contributing to the cost and sustainability of ^61^Cu production. The first is the cost of the target material, especially when enriched isotopes are used. Although upscaling production is possible only with enriched isotopes, their higher cost and limited supply require target material recycling to minimize expenses.

A second factor is the operation and maintenance costs of the cyclotron. These costs directly impact the cost per hour of irradiation. Limiting the irradiation time and complexity of the production can be beneficial in contract manufacturing to minimize costs.

A third factor are the separation and purification costs, as specialized processes are required to isolate the desired ^61^Cu from the target material and other by-products. These processes require expensive trace-metal–free chemicals and consumables.

A final factor is radiopharmaceutical production and distribution: additional costs are involved in the final product’s formulation, manufacturing, and distribution. With its relatively long half-life, ^61^Cu provides a distribution range of over 400 km and the advantages of centralized manufacturing and longer shelf-life than for ^68^Ga or ^18^F radiopharmaceuticals.

## CONCLUSION

Using cyclotron-produced ^61^Cu offers the advantage of streamlined production and logistics similar to centralized ^18^F production. Furthermore, it allows for quick and convenient cold kit radiolabeling, provides the potential for theranostics using the companion therapeutic ^67^Cu via chelator-based radiochemistry, and is a sustainable and cost-effective approach. So far, only 1 pilot clinical study with [^61^Cu]Cu-ATSM for imaging hypoxia (NCT00585117) has been registered (in 2008), but there are no data available. Clinical studies with ^61^Cu-labeled somatostatin and PSMA analogs are planned in 2024 that may indicate the role of ^61^Cu in clinical PET imaging. In the emerging era of radiopharmaceuticals and radiotheranostics, ^61^Cu radiotracers are a valuable alternative. Still, their future adoption as a preferred choice is yet to be determined.

## DISCLOSURE

Melpomeni Fani is a scientific advisor of Nuclidium AG and coinventor on 2 patent applications filed by Nuclidium AG and the University of Basel, Switzerland. No other potential conflict of interest relevant to this article was reported.
